# Compulsory drug detention exposure is associated with not receiving antiretroviral treatment among people who inject drugs in Bangkok, Thailand: a cross-sectional study

**DOI:** 10.1186/s13011-015-0013-6

**Published:** 2015-05-06

**Authors:** Kanna Hayashi, Lianping Ti, Anchalee Avihingsanon, Karyn Kaplan, Paisan Suwannawong, Evan Wood, Julio S G Montaner, Thomas Kerr

**Affiliations:** British Columbia Centre for Excellence in HIV/AIDS, 608-1081 Burrard Street, Vancouver, BC V6Z 1Y6, Canada; Department of Medicine, Faculty of Medicine, University of British Columbia, 317-2194 Health Sciences Mall, Vancouver, BC V6T 1Z3, Canada; School of Population and Public Health, University of British Columbia, 2206 East Mall, Vancouver, BC V6T 1Z3, Canada; HIV-NAT, Thai Red Cross AIDS Research Centre, 104 Ratchadamri Rd, Prathumwan, Bangkok, 10300 Thailand; Department of Medicine, Faculty of Medicine, Chulalongkorn University, 254 Phayathai Road, Bangkok, 10330 Thailand; Thai AIDS Treatment Action Group, 18/89 Vipawadee Rd,soi 40 Chatuchak, Bangkok, 10900 Thailand

**Keywords:** ART, Injection drug use, Compulsory drug detention, Peer-based intervention, Thailand

## Abstract

**Background:**

Thailand has experienced a longstanding epidemic of HIV among people who inject drugs (PWID). However, antiretroviral treatment (ART) coverage among HIV-positive PWID has historically remained low. While ongoing drug law enforcement involving periodic police crackdowns is known to increase the risk of HIV transmission among Thai PWID, the impact of such drug policy approaches on the ART uptake has been understudied. Therefore, we sought to identify factors associated with not receiving ART among HIV-positive PWID in Bangkok, Thailand, with a focus on factors pertaining to drug law enforcement.

**Methods:**

Data were collected from a community-recruited sample of HIV-positive PWID in Bangkok who participated in the Mitsampan Community Research Project between June 2009 and October 2011. We identified factors associated with not receiving ART at the time of interview using multivariate logistic regression.

**Results:**

In total, 128 HIV-positive PWID participated in this study, with 58 (45.3%) reporting not receiving ART at the time of interview. In multivariate analyses, completing less than secondary education (adjusted odds ratio [AOR]: 3.32 ; 95% confidence interval [CI]: 1.48 – 7.45), daily midazolam injection (AOR: 3.22, 95% CI: 1.45 – 7.15) and exposure to compulsory drug detention (AOR: 3.36, 95% CI: 1.01 – 11.21) were independently and positively associated with not receiving ART. Accessing peer-based healthcare information or support services was independently and positively associated with receiving ART (AOR: 0.21, 95% CI: 0.05 – 0.84).

**Conclusions:**

Approximately half of our study group of HIV-positive PWID reported not receiving ART at the time of interview. Daily midazolam injectors, those with lower education attainment, and individuals who had been in compulsory drug detention were more likely to be non-recipients of ART whereas those who accessed peer-based healthcare-related services were more likely to receive ART. These findings suggest a potentially adverse impact of compulsory drug detention and highlight the need to expand interventions to facilitate access to ART among HIV-positive PWID in this setting.

## Background

In many parts of the world, people who inject drugs (PWID) are severely affected by HIV/AIDS [1]. It is estimated that injection drug use accounts for more than one-quarter of new HIV infections outside of sub-Saharan Africa [2]. Injection drug use-driven HIV epidemics are particularly salient in Asia, which accommodates seven of the 15 countries worldwide where >100,000 PWID reside and where an estimated HIV prevalence among PWID is >10% [3]. Although antiretroviral treatment (ART) has dramatically reduced HIV-related morbidity and mortality [4,5], and has recently been shown to prevent HIV transmission [6], access to ART is disproportionately low among PWID in many settings [7].

Recent reviews have suggested various factors constraining access to ART among PWID worldwide, including individual (e.g., ongoing drug use), social (e.g., stigma), and structural factors (e.g., incarceration) [8,9]. In particular, barriers stemming from punitive drug policies have drawn increasing attention [8,9]. Although recent international research has elucidated the adverse effects of incarceration on access and adherence to ART [8,10], other impacts associated with the criminalization of drug use have not been fully investigated. In particular, some Asian countries, including Thailand, continue to operate systems of compulsory drug detention [11-13], despite twelve United Nations agencies calling on governments to abolish such systems due to the associated human rights violations and substandard addiction treatment [14]. In Thailand, the number of people who use drugs detained amounted to more than 102,000 in 2011 [15].

Thailand has experienced a longstanding HIV epidemic among PWID, with an estimated HIV prevalence in this population ranging between 30–50% for more than two decades [16,17]. Since 2000, Thailand has developed a national initiative to provide ART for free or at a reduced cost [18]. As a result, in 2011, 65% of eligible people living with HIV (PLHIV) reportedly received ART, which stands as a relatively high level of coverage for a middle-income country [17,19]. In contrast, only 2 per 100 HIV-positive PWID were estimated to have ever accessed ART in Thailand in 2007 [7], although in our 2009 study, 45% of 67 HIV-positive PWID in Bangkok reported receiving ART at the time of interview [20].

The Thai government has for many years implemented drug prohibition approaches involving periodic police crackdowns [21]. While some potentially effective measures to improve access to ART among HIV-positive PWID exist in this setting, including methadone treatment and peer-support groups [8], few studies have investigated factors that promote or undermine uptake of ART among PWID within a context of drug law enforcement involving periodic police crackdowns. Therefore, we sought to identify factors associated with not receiving ART among a community-recruited sample of HIV-positive PWID in Bangkok, Thailand, with a focus on factors pertaining to drug law enforcement.

## Methods

### Study sample

Data were derived from the Mitsampan Community Research Project, a collaborative research effort involving the Mitsampan Harm Reduction Center (MSHRC; a drug user-run drop-in centre in Bangkok, Thailand), Thai AIDS Treatment Action Group (Bangkok, Thailand), Chulalongkorn University (Bangkok, Thailand), and the British Columbia Centre for Excellence in HIV/AIDS/University of British Columbia (Vancouver, Canada). This serial cross-sectional study aims to investigate drug-using behaviour, healthcare access, and other drug-related harms among PWID in Bangkok. Between June 2009 and October 2011, the research partners undertook two waves of surveying, which involved an accumulated total of 757 community-recruited PWID in Bangkok. Potential participants were recruited through peer outreach efforts and word-of-mouth, and were invited to attend the MSHRC or O-Zone House (another drop-in centre in Bangkok) in order to be part of the study. Recruitment criteria included adults residing in Bangkok or in adjacent provinces who had injected drug(s) in the past six months. All participants provided informed consent and completed an interviewer-administered questionnaire eliciting a range of information, including demographic characteristics, drug use patterns, HIV serostatus, and experiences with drug law enforcement and accessing healthcare. Upon completion of the questionnaire, participants received a stipend of 350 Thai Baht (approximately US$12). The study was approved by the research ethics boards at Chulalongkorn University (COA No. 085/2009, 093/2011) and the University of British Columbia (H08-00702, H11-00581).

All participants who completed the interview in 2009 or 2011 and reported being HIV-positive were eligible for inclusion in this study. Given that some individuals were interviewed in both 2009 and 2011, we included all participants from the first wave and only new participants from the second wave in order to ensure the independence of the observations analyzed in this study. The sample of each survey wave was further restricted to individuals who had complete data for the present analyses.

### Variable selection

The primary outcome of interest was not receiving ART at the time of interview, defined as answering “No” to the following question: “Are you currently taking antiretrovirals (ARVs)?” Based on previous literature [8], a set of explanatory variables were hypothesized to be associated with the outcome. Demographic characteristics included median age (≥38 years *vs.* < 38 years); gender (female *vs.* male); and education attainment (< secondary education *vs.* ≥ secondary education). Indicators for the severity of substance use included: heroin injection (daily *vs.* < daily); methamphetamine injection (daily *vs.* < daily); midazolam (a short-acting benzodiazepine) injection (daily *vs.* < daily); and alcohol consumption (daily *vs.* < daily). Exposure to drug law enforcement included: ever beaten by police; ever incarcerated; and ever in compulsory drug detention. Finally, experiences with accessing healthcare included: ever accessed methadone treatment; ever received healthcare information or support services at the MSHRC; and reporting barriers to healthcare (any *vs.* none). As in our previous work [22], our barriers to healthcare variable included a range of potential barriers, including but not limited to: long wait times, poor treatment by healthcare providers, financial barriers, and transportation issues. All variables were coded dichotomously as yes *vs.* no, unless otherwise stated. Variables related to drug use referred to the previous six months.

### Statistical analyses

To examine bivariate associations between the outcome and the explanatory variables of interest, we used the Pearson *X*^*2*^ test. Fisher’s exact test was used when one or more of the cells contained expected values less than or equal to five. Three variables (i.e., methamphetamine injection, alcohol consumption, and ever incarcerated) met this criterion. Next, we used an *a priori*-defined statistical protocol that examined factors associated with the outcome by fitting a multivariate logistic regression model that included all variables that were significantly associated with the outcome at the *p* < 0.05 level in bivariate analyses. All *p*-values were two-sided. All statistical analyses were performed using SAS software version 9.3 (SAS, Cary, NC).

## Results

**Figure 1:**
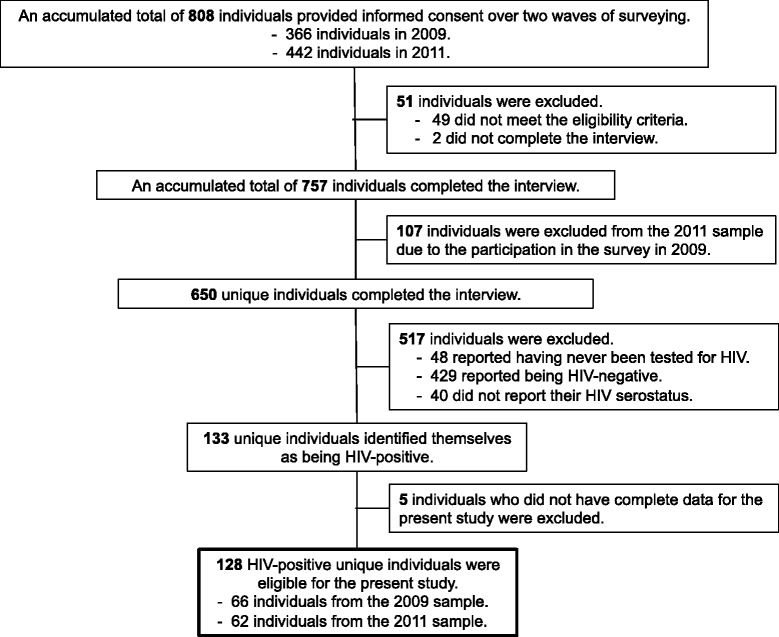
Determination of the analytic sample.

Figure 1 describes the determination of the analytic sample. As shown, 133 (20.5%) of 650 unique individuals who completed the interview over the two waves of surveying in 2009 and 2011 identified themselves as being HIV-positive. Among the remaining 517 participants who did not report being HIV-positive, 336 (65.0%) reported having been tested for HIV in the previous six months. Of the 133 HIV-positive participants, 5 (3.8%) were excluded from the present analysis due to incomplete data. Therefore, a total of 128 HIV-positive participants were eligible for the present study.

Among 128 HIV-positive PWID participated in this study, 25 (19.5%) were women, and the median age was 38 years (interquartile range: 34 – 44 years). In total, 58 (45.3%) individuals reported not receiving ART at the time of interview. Of these, 36 (62.1%) reported having not seen an HIV doctor on a regular basis (i.e., at least once in six months).

Table 1 shows the summary statistics of the sample and the results of bivariate analyses. As shown, in bivariate analyses, completing less than secondary education (odds ratio [OR]: 3.54; 95% confidence interval [CI]: 1.70 – 7.39), daily midazolam injection in the previous six months (OR: 3.64; 95% CI: 1.75 – 7.58) and having ever been in compulsory drug detention (OR: 3.08; 95% CI: 1.09 – 8.72) were significantly and positively associated with not receiving ART. Having ever received healthcare information or support services at the MSHRC was significantly and negatively associated with not receiving ART (OR: 0.22; 95% CI: 0.06 – 0.80).

**Table 1:** **Bivariate associations with not receiving ART among HIV-positive PWID in Bangkok, Thailand (** ***n*** **= 128)**

**Characteristic**	**Currently on ART**		**Odds ratio (95%CI)**	***p -*** **value**
	**No 58 (45.3%)**	**Yes 70 (54.7%)**		
***Demographic***				
**Older age**				
≥38 years old	30 (51.7%)	35 (50.0%)	1.07 (0.53 – 2.15)	0.846
<38 years old	28 (48.3%)	35 (50.0%)		
**Gender**				
Female	12 (20.7%)	13 (18.6%)	1.14 (0.48 – 2.75)	0.764
Male	46 (79.3%)	57 (81.4%)		
**Education attainment**				
< Secondary education	34 (58.6%)	20 (28.6%)	3.54 (1.70 – 7.39)	<0.001
≥ Secondary education	24 (41.4%)	50 (71.4%)		
***Substance use behaviour***				
Heroin injection*				
Daily	9 (15.5%)	8 (11.4%)	1.42 (0.51 – 3.96)	0.497
< Daily	49 (84.5%)	62 (88.6%)		
Methamphetamine injection*				
Daily	7 (12.1%)	3 ( 4.3%)	3.07 (0.65 – 19.10)	0.184^†^
< Daily	51 (87.9%)	67 (95.7%)		
Midazolam injection*				
Daily	38 (65.5%)	24 (34.3%)	3.64 (1.75 – 7.58)	<0.001
< Daily	20 (34.5%)	46 (65.7%)		
Alcohol consumption*				
Daily	3 ( 5.3%)	3 ( 4.3%)	1.24 (0.16 – 9.63)	>0.999^†^
< Daily	54 (94.7%)	67 (95.7%)		
***Experiences with drug law enforcement***				
**Ever beaten by police**				
Yes	33 (56.9%)	28 (40.0%)	1.98 (0.98 – 4.01)	0.057
No	25 (43.1%)	42 (60.0%)		
**Ever incarcerated**				
Yes	56 (96.6%)	63 (90.0%)	3.11 (0.56 – 31.65)	0.182^†^
No	2 ( 3.4%)	7 (10.0%)		
**Ever in compulsory drug detention**				
Yes	13 (22.4%)	6 ( 8.6%)	3.08 (1.09 – 8.72)	0.028
No	45 (77.6%)	64 (91.4%)		
***Healthcare access***				
**Ever accessed methadone treatment**				
Yes	51 (87.9%)	62 (88.6%)	0.94 (0.32 – 2.77)	0.911
No	7 (12.1%)	8 (11.4%)		
**Ever received healthcare information or support services at the MSHRC**				
Yes	3 ( 5.2%)	14 (20.0%)	0.22 (0.06 – 0.80)	0.014
No	55 (94.8%)	56 (80.0%)		
Reporting barriers to healthcare*				
Any	33 (56.9%)	48 (68.6%)	0.61 (0.29 – 1.25)	0.173
None	25 (43.1%)	22 (31.4%)		

PWID: people who inject drugs; ART: antiretroviral treatment; CI: confidence interval; MSHRC: Mitsampan Harm Reduction Center.

*denotes activities in the previous 6 months.

^†^Fisher’s exact test was used.

Table 2 shows the results of the multivariate analysis. As shown, completing less than secondary education (adjusted odds ratio [AOR]: 3.32; 95% CI: 1.48 – 7.45), daily midazolam injection (AOR: 3.22; 95% CI: 1.45 – 7.15) and having ever been in compulsory drug detention (AOR: 3.36; 95% CI: 1.01 – 11.21) remained independently and positively associated with not receiving ART. Having ever received healthcare information or support services at the MSHRC also remained independently and negatively associated with the outcome (AOR: 0.21; 95% CI: 0.05 – 0.84).

**Table 2:** **Multivariate logistic regression analysis of factors associated with not receiving ART among HIV-positive PWID in Bangkok, Thailand (** ***n*** **= 128)**

**Characteristic**	**Adjusted odds ratio (95% CI)**	***p*** **-value**
**Education attainment**		
(< Secondary education *vs.* ≥ Secondary education)	3.32 (1.48 – 7.45)	0.004
Midazolam injection*		
(Daily *vs.* < Daily)	3.22 (1.45 – 7.15)	0.004
**Ever in compulsory drug detention**		
(Yes *vs.* No)	3.36 (1.01 – 11.21)	0.049
**Ever received healthcare information or support services at the MSHRC**		
(Yes *vs.* No)	0.21 (0.05 – 0.84)	0.028

PWID: people who inject drugs; ART: antiretroviral treatment; CI: confidence interval; MSHRC: Mitsampan Harm Reduction Center.

*denotes activities in the previous 6 months.

## Discussion

We found that approximately half of our study group of HIV-positive PWID in Bangkok reported not receiving ART. Because blood specimens were not collected, we were unable to assess the indication for ART initiation (e.g., CD4 counts <350 cells/mm^3^ according to the 2010 Thai national guidelines for ART [19]) among those not receiving ART at the time of interview. However, the majority of Thai PLHIV have been shown to be diagnosed with HIV infection at a late stage of HIV disease, with approximately 60% starting ART with CD4 levels of <100 cells per cubic millimeter [17], and therefore a substantial portion of our sample of untreated HIV-positive PWID may have met ART eligibility criteria. Future research should investigate stages of HIV disease among HIV-positive PWID who are not receiving ART. Nonetheless, given the persistently high prevalence of syringe sharing among HIV-positive PWID in this setting [17,20,23], and the now widely recognized impact of HIV treatment on the prevention of new HIV infections [24], ensuring access to ART among PWID remains a high priority in Thailand’s response to the HIV epidemic [17]. In this regard, our findings provide important insights into barriers to and facilitators of ART access among HIV-positive PWID in Bangkok. In addition, we note that evolving ART guidelines have dramatically increased the proportion of PLHIV in need of ART, particularly among high-risk populations marked with significant comorbidities, including PWID, as the Joint United Nations Programme on HIV/AIDS has recently estimated that approximately 85% of PLHIV are eligible for ART provision under the 2013 World Health Organization criteria [25]. Furthermore, beginning in October 2014, the Thai national ART guidelines has removed CD4 count from the ART eligibility criteria, and now all PLHIV in Thailand are eligible for ART initiation regardless of CD4 count [23].

Of particular concern is the independent association between exposure to compulsory drug detention and not receiving ART. In Thailand, it has been reported that some public hospitals collect and share information about individual drug use with police [26]. Given the punitive nature of compulsory drug detention centres [21], our findings may suggest that HIV-positive PWID who have been detained in such centres are reluctant to access HIV treatment due to fear of disclosing their drug use to healthcare providers and thereby risk being readmitted to detention centres. Consistent with our interpretation, a recent study also found an independent relationship between exposure to compulsory drug detention and the avoidance of healthcare among PWID in this setting [21]. Furthermore, a previous report also suggested inconsistent availability of ART across the custodial settings as well as a lack of continuity of healthcare, including ART, on entry to and on release from detention in Thailand [26]. These findings suggest that compulsory drug detention may be placing an undue burden on public health by undermining ex-detainees’ access to HIV treatment. Future research should longitudinally assess the impact of compulsory drug detention exposure on HIV disease progression in this setting.

The finding that HIV-positive PWID who accessed peer-based healthcare information and support services were more likely to be on ART is congruent with a large body of literature indicating the effectiveness of peer-based interventions in providing HIV/AIDS education and supporting access to HIV care among PWID [8,27]. Our findings that the positive effect of peer-based support services on the ART uptake remained significant even after adjusting for the effect of education attainment further suggest the effectiveness of such peer-based interventions. Since 1992, the Thai Ministry of Public Health has supported many PLHIV peer support groups, which have been working to facilitate access to HIV treatment among PLHIV in the country [28]. However, until recently, there have been few PWID-specific PLHIV peer support services, and those that exist are primarily funded by international donors [29]. The MSHRC is one of those few sites that provide a variety of services via a peer-delivered approach, including sterile syringe distribution, harm reduction education, food and drinks, and support for healthcare access [30]. A previous study has shown that this model has successfully reached sub-populations of PWID who were particularly vulnerable to HIV infection and other drug-related harm in this setting [30]. Given the profound stigma against PWID in healthcare settings in Thailand [26,31], the expansion of PWID-specific peer support, such as that offered at the MSHRC, may be crucial for facilitating access to ART among HIV-positive PWID in this setting.

We also found that daily midazolam injection was independently associated with not receiving ART. Midazolam is a short-acting benzodiazepine that can be legally obtained through private clinics in Bangkok [32]. While injection of benzodiazepines is common among opioid users in many settings [33,34], in Bangkok, midazolam is the most commonly injected drug among PWID [22]. Amnesia and severe injection-related injuries and disease associated with midazolam use [22] indicate a need for additional support services if these daily midazolam injectors are to initiate ART.

This study has several limitations. First, we cannot infer causation from this observational study. Further, the cross-sectional study design did not allow us to assess temporal relationships between the outcome and explanatory variables. Second, due to the lack of HIV-related clinical data (e.g., CD4 counts), we were unable to assess the eligibility for ART among our sample. While we also recognize that it would have been ideal to utilize a more sensitive assessment of untreated HIV infection (e.g., whether a participant was not receiving ART at the time of interview due to poor adherence, treatment discontinuation, or being ineligible for ART, etc.), we were unable to include such measurements in our questionnaire. Future research should seek to use a refined measure of untreated HIV infection. To this end, an exploratory qualitative study to understand reasons for not accessing ART would provide useful data for refining the measure and selecting the study variables. Third, the self-reported data may have been affected by some reporting biases, including socially desirable responding and recall bias. For example, these biases might have led to the underestimation of the prevalence of compulsory drug detention exposure (due to socially desirable reporting) and ever receiving services at the MSHRC (due to recall bias). However, we believe that it is unlikely that such information biases differentially influenced the data by HIV treatment status. We also note that this type of self-reported data has been commonly utilized in observational studies involving PWID and has been found to be valid [35]. Lastly, due to the small sample size, there were wide intervals around some of the estimates reported. Also, as the study sample was not randomly selected, our findings may not be generalizable to PWID populations in Thailand or elsewhere.

## Conclusions

In summary, we found that about half of our study group of HIV-positive PWID in Bangkok reported that they were not receiving ART at the time of interview. Daily midazolam injectors, those with lower education attainment, and individuals who had been in compulsory drug detention were more likely to be non-recipients of ART. In contrast, individuals who accessed peer-based healthcare information and support services were more likely to be on ART. These findings suggest a potentially adverse impact of compulsory drug detention and indicate a need for expanding interventions to facilitate access to ART among HIV-positive PWID in this setting.

## Abbreviations

ART: Antiretroviral treatment
HIV: Human immunodeficiency virus
MSHRC: Mitsampan Harm Reduction Center
PLHIV: People living with HIV
PWID: People who inject drugs

## References

[1] Mathers BM, Degenhardt L, Phillips B, Wiessing L, Hickman M, Strathdee SA (2008). Global epidemiology of injecting drug use and HIV among people who inject drugs: a systematic review. Lancet.

[2] Joint United Nations Programme on HIV/AIDS (2014). The gap report. Joint United Nations Program on HIV/AIDS.

[3] Joint United Nations Programme on HIV/AIDS (2010). HIV in Asia and the Pacific: getting to zero.

[4] Hogg RS, O'Shaughnessy MV, Gataric N, Yip B, Craib K, Schechter MT (1997). Decline in deaths from AIDS due to new antiretrovirals. Lancet.

[5] The Antiretroviral Therapy in Lower Income Countries ART-LINC Collaboration, ART Cohort Collaboration ART-CC groups (2011). Mortality of HIV-1-infected patients in the first year of antiretroviral therapy: comparison between low-income and high-income countries. Lancet.

[6] Cohen MS, Chen YQ, McCauley M, Gamble T, Hosseinipour MC, Kumarasamy N (2011). Prevention of HIV-1 infection with early antiretroviral therapy. N Engl J Med.

[7] Mathers BM, Degenhardt L, Ali H, Wiessing L, Hickman M, Mattick RP (2010). HIV prevention, treatment, and care services for people who inject drugs: a systematic review of global, regional, and national coverage. Lancet.

[8] Wolfe D, Carrieri MP, Shepard D (2010). Treatment and care for injecting drug users with HIV infection: a review of barriers and ways forward. Lancet.

[9] Milloy MJ, Montaner J, Wood E (2012). Barriers to HIV treatment among people who use injection drugs: implications for “treatment as prevention”. Curr Opin HIV AIDS.

[10] Milloy MJ, Kerr T, Buxton J, Rhodes T, Guillemi S, Hogg R (2011). Dose–response effect of incarceration events on nonadherence to HIV antiretroviral therapy among injection drug users. J Infect Dis.

[11] Pearshouse R (2009). Compulsory drug treatment in Thailand: observations on the Narcotic Addict Rehabilitation Act B.E. 2545 (2002).

[12] Cohen JE, Amon JJ (2008). Health and human rights concerns of drug users in detention in Guangxi Province. China PLoS Med.

[13] Wolfe D, Saucier R (2010). In rehabilitation's name? Ending institutionalised cruelty and degrading treatment of people who use drugs. Int J Drug Policy.

[14] International Labour Organisation, Office of the High Commissioner for Human Rights, United Nations Development Programme, United Nations Educational, Scientific and Cultural Organization, United Nations Population Fund, United Nations High Commissioner for Refugees, et al. Joint statement: compulsory drug detention and rehabilitation centres. 2012. http://www.unaids.org/sites/default/files/en/media/unaids/contentassets/documents/document/2012/JC2310_Joint%20Statement6March12FINAL_en.pdf. Accessed 1 Apr 2015.

[15] Office of the Narcotics Control Board of Thailand (2011). Thailand narcotics control annual report 2011.

[16] National AIDS Prevention and Alleviation Committee. UNGASS country progress report Thailand: reporting period: January 2008 - December 2009. 2010. http://data.unaids.org/pub/Report/2010/thailand_2010_country_progress_report_en.pdf. Accessed 1 Apr 2015.

[17] Thai National AIDS Committee. 2012 Thailand AIDS response progress report. 2013. http://www.unaids.org/sites/default/files/en/dataanalysis/knowyourresponse/countryprogressreports/2012countries/ce_th_narrative_report.pdf. Accessed 1 Apr 2015.

[18] Chasombat S, McConnell MS, Siangphoe U, Yuktanont P, Jirawattanapisal T, Fox K (2009). National expansion of antiretroviral treatment in Thailand, 2000–2007: program scale-up and patient outcomes. J Acquir Immune Defic Syndr.

[19] Sungkanuparph S, Techasathit W (2011). Thai national guidelines for antiretroviral therapy in HIV-1 infected adults and adolescents 2010. Asian Biomed.

[20] Hayashi K, Montaner J, Kaplan K, Suwannawong P, Wood E, Qi J (2011). Low uptake of hepatitis C testing and high prevalence of risk behavior among HIV-positive injection drug users in Bangkok. Thailand J Acquir Immune Defic Syndr.

[21] Kerr T, Hayashi K, Ti L, Kaplan K, Suwannawong P, Wood E (2014). The impact of compulsory drug detention exposure on the avoidance of healthcare among injection drug users in Thailand. Int J Drug Policy.

[22] Hayashi K, Suwannawong P, Ti L, Kaplan K, Wood E, Kerr T (2013). High rates of midazolam injection and associated harms in Bangkok. Thailand Addiction.

[23] Thai National AIDS Committee. 2014 Thailand AIDS response progress report. 2014. http://www.unaids.org/sites/default/files/country/documents//THA_narrative_report_2014.pdf. Accessed 1 Apr 2015.

[24] Cohen MS, Smith MK, Muessig KE, Hallett TB, Powers KA, Kashuba AD (2013). Antiretroviral treatment of HIV-1 prevents transmission of HIV-1: where do we go from here?. Lancet.

[25] Joint United Nations Programme on HIV/AIDS (2014). Global AIDS response progress reporting 2014: construction of core indicators for monitoring the 2011 United Nations Political Declaration on HIV and AIDS.

[26] Human Rights Watch (2007). Thai AIDS Treatment Action Group. Deadly denial: barriers to HIV/AIDS treatment for people who use drugs in Thailand.

[27] Needle RH, Burrows D, Friedman SR, Dorabjee J, TouzÈ G, Badrieva L (2005). Effectiveness of community-based outreach in preventing HIV/AIDS among injecting drug users. Int J Drug Policy.

[28] Chasombat S, Lertpiriyasuwat C, Thanprasertsuk S, Suebsaeng L, Lo YR (2006). The national access to antiretroviral program for PHA (NAPHA) in Thailand. Southeast Asian J Trop Med Public Health.

[29] Stoicescu C, Harm Reduction International (2012). The global state of harm reduction 2012: towards an integrated response.

[30] Kerr T, Hayashi K, Fairbairn N, Kaplan K, Suwannawong P, Zhang R (2010). Expanding the reach of harm reduction in Thailand: Experiences with a drug user-run drop-in centre. Int J Drug Policy.

[31] Chan KY, Stoove MA, Reidpath DD (2008). Stigma, social reciprocity and exclusion of HIV/AIDS patients with illicit drug histories: a study of Thai nurses' attitudes. Harm Reduct J.

[32] Kiatying-Angsulee N, Kulsomboon V, Kittisopee T, Patcharapisarn N, Sriwiriyanuparb W, Sirisinsuk Y, et al. Midazolam use in injecting drug users (IDUs) in Bangkok: preliminary results of a qualitative study. The 15^th^ International AIDS Conference. Bangkok, Thailand; 2004. Abstractno.WePeC6048.

[33] Lintzeris N, Nielsen S (2010). Benzodiazepines, methadone and buprenorphine: interactions and clinical management. Am J Addict.

[34] Darke S (1994). Benzodiazepine use among injecting drug users: problems and implications. Addiction.

[35] Darke S (1998). Self-report among injecting drug users: a review. Drug Alcohol Depend.

